# Growth and survival of *Bifidobacterium breve* and *Bifidobacterium longum* in various sugar systems with fructooligosaccharide supplementation

**DOI:** 10.1007/s13197-022-05361-z

**Published:** 2022-01-20

**Authors:** Priyanka Parhi, Keang Peng Song, Wee Sim Choo

**Affiliations:** School of Science, Monash University Malaysia, 47500 Bandar Sunway, Selangor Malaysia

**Keywords:** Glucose, Fructose, Lactose, Sucrose, Prebiotic, Probiotic

## Abstract

This study aims to investigate the effect of fructooligosaccharide (FOS) (0.5, 1, 2, 3, and 4%) supplementation on the growth and survival of *Bifidobacterium breve* and *Bifidobacterium longum* in glucose, fructose, lactose, and sucrose (2, 3, and 4%) systems with 24-h growth and 10-day survival assays at 37 °C. FOS supplementation showed a higher growth-promoting effect on *B. longum* than *B. breve* in various sugar systems. The highest percentage of increase in growth index, 78.5%, was observed with 4% sucrose supplemented with 0.5% FOS in *B. longum*. In comparison, the highest percentage increase in growth index, 5.6 and 6.6%, was observed in the presence of 2% glucose and 4% lactose supplemented with 0.5% FOS in *B. breve*. In survival assay, FOS supplementation (0.5–4%) in a 2% lactose system showed the highest positive effect on the cell viability of *B. longum* on day-10. As for *B. breve*, FOS supplementation (1 and 2%) in the 2% sucrose system showed the highest positive effect on the cell viability, followed by FOS supplementation (0.5, 3, and 4%) in 2% sucrose and FOS supplementation (3 and 4%) in 2% lactose on day-10. This study demonstrated that the efficacy of FOS supplementation was depended on its concentration, sugar system and its concentration, and *Bifidobacterium* strain.

**Supplementary Information:**

The online version contains supplementary material available at 10.1007/s13197-022-05361-z.

## Introduction

Probiotics help normalize perturbed microbiota, organic acid, specially lactis acid and short-chain fatty acids production, regulation of intestinal transit, and competitive exclusion of pathogens. The current definition of probiotics, “live microorganisms that, when administered in adequate amounts, confer a health benefit on the host,” was provided by International Scientific Association for Probiotics and Prebiotics (ISAPP) in 2014 (Hill et al. 2014). The natural ability of bifidobacteria to survive in the gastrointestinal tract, resistance mechanisms to bile’s salts, and unique fructose-6-phosphate pathway to ferment hexoses make them one of the widely used probiotic bacteria (Shah 2011). *Bifidobacterium*, a major bacterial group, are classified as gram-positive, non-spore-forming, non-motile, and catalase-negative anaerobes (Shah 2011). They are common inhabitants of the gastrointestinal tract of humans, and vaginal walls account for up to 25% of the total culturable gut microorganisms in adults. *Bifidobacterium* helps in plant polysaccharide digestion, human pathogen growth inhibition, resists bile salts, influences hosts' fatty acid metabolism, and shows antipathogenic, anti-inflammatory, and immunomodulation activities (Fanning et al. 2012).

Fructooligosaccharides (FOS) are non-digestible carbohydrates that represent one of the significant classes of bifidogenic oligosaccharides, which are extracted from plants such as yacon root, asparagus, sugar beet, garlic, chicory roots, leeks, onion, Jerusalem artichoke, tomato, and wheat, rye, or barley (Flamm et al. 2001). The fructose units in this mixture of linear fructose polymers and oligomers are linked by β (2–1) bonds with terminal glucose (Flamm et al. 2001). Therefore, they are not hydrolyzed by human digestive enzymes and are not absorbed into the gastrointestinal tract (Gibson et al. 2004). Prebiotics should not get absorbed by the upper gastrointestinal tract and should resist acid hydrolysis or any modifications by digestive enzymes (Roberfroid 2002). FOS is one of the established prebiotics, defined as “a substrate that is selectively utilized by host microorganisms conferring a health benefit” by the ISAPP (Gibson et al. 2017).

Recently ISAPP redefined synbiotic as “a mixture comprising live microorganisms and substrate(s) selectively utilized by host microorganisms that confers a health benefit on the host” (Swanson et al. 2020). Synbiotics were developed and researched to overcome possible survival difficulties for probiotics during production, storage, and passage through the gastrointestinal tract, significantly improving the probiotic effect (Swanson et al. 2020). However, the supplementation of FOS showed inconsistent effects in various food systems. For examples, FOS supplementation in skim milk improved the viability of *B. bifidum* (Shin et al. 2000) and *B. longum* (Choi and Shin 2006). Similar positive effect of FOS supplementation was observed on the growth of *B. lactis* in fermented milk (Oliveira et al. 2009), *B. longum* FTDC 8643 in soymilk (Yeo and Liong 2010), and *B.* *adolescentis* and *B.* *catenulatum* in milk (Padalino et al. 2012). In addition, Akalın et al. (2004) reported that FOS supplementation in yogurt resulted in better stability of *B. animalis* than *B. longum* during 21-day storage at 4 °C. Similarly, Celestin et al (2015) reported that FOS supplementation in goat milk yogurts resulted in a higher cell count of *L. acidophilus* than *B. bifidum*. On the other hand, no effect of FOS supplementation was reported on the growth of *B. infantis* in fermented milk (Basholli-Salihu et al. 2013), *B. animalis* subsp. *lactis* in fermented cream cheese (Speranza et al. 2018) and *B. animalis* Bb-12 in soymilk (Battistini et al. 2018). Although there were studies that showed no positive effect of prebiotic supplementation on the viability of probiotics during food fermentation, the use of prebiotics should not be deterred as the positive effect might be exerted in the human large intestine (Amanda and Choo 2018). Since the beneficial effects of prebiotic supplementation on the growth and viability of probiotics in complex food matrices are inconclusive, this study proposed that the effect of prebiotic like FOS on probiotics varies depending on its concentration and the type and concentration of sugar.

Glucose and fructose are monosaccharides that are predominant sugars found in vegetables, fruits, and grains such as rice grain, barley, corn, honey, red grapes, apples, and pomegranates (Liu et al. 2006; White 2014). Lactose, a disaccharide of galactose and glucose with β-1,4-glycosidic bond, is the predominant sugar in animal milk and animal milk products (Fox 2011; Turroni et al. 2011). Similarly, sucrose, a disaccharide of glucose and fructose with α-1, β-2-glycosidic bond, is the table sugar derived from sugar beet or sugar cane (Liu et al. 2006; White 2014). Bifidobacterial genomes encode several carbohydrate kinds of transport and modifying enzymes that allow bifidobacteria to utilize and grow on different carbohydrates (Mazé et al. 2007; Parche et al. 2007). *B. breve* and *B. longum* are clinically well studied for their probiotical effects on human and animal health and are included in multi-organism probiotics. The potential of *B. breve* and *B. longum* to efficiently digest plant polysaccharides, resistance to low pH and bile salts, influence host fatty acid metabolism, and show antipathogenic, anti-inflammatory, and immunomodulation activities made them widespread probiotics (Sgorbati et al. 1995; Fanning et al. 2012). To provide insight on food formulations containing stable counts of bifidobacteria, the present study aimed to investigate the effects of FOS supplementation at various concentrations on the growth and survival of *Bifidobacterium breve* and *Bifidobacterium longum* in different concentrations of four sugar systems, namely glucose, fructose, sucrose, and lactose.

## Materials and methods

### Microbial culture and reactivation of probiotic culture

*Bifidobacterium longum* (ATCC BAA-999), and *Bifidobacterium breve* (ATCC 15700) were purchased directly from American Type Culture Collection (ATCC) Manassas VA, U.S.A. *B. longum* and *B. breve* was activated from a glycerol stock. *Bifidobacterium* agar media was purchased from HiMedia, India. *B. breve* and *B. longum* were maintained in standard *Bifidobacterium* agar medium at pH 5.5 ± 0.2*.*

During inoculum preparation, *B. breve* and *B. longum* were grown in *Bifidobacterium* broth for 18 h at 37 °C, 120 rpm incubator shaker under anaerobic condition followed by centrifugation at 10,000 × *g* for 10 min at 4˚C.

### Preparation of modified *Bifidobacterium* broth for *B. longum* and *B. breve*

Modified *Bifidobacterium* broth (1000 mL) with pH 6.5 ± 0.2 was prepared using peptone special (22.2 g), NaCl (4.8 g), and L-cysteine hydrochloride monohydrate (0.5 g) with prebiotics and sugars. Four different sugars (fructose, sucrose, glucose, and lactose) at different concentrations (1, 2, 3, and 4%) with FOS supplementation at different concentrations (0.5, 1, 2, 3, and 4%) were used for growth and survival assays. Agar powder, phosphate-buffered saline (PBS) tablets, anaerogens, peptone special, sodium chloride (NaCl), and L-cysteine hydrochloride monohydrate were purchased from Oxoid, UK. Glucose, fructose, sucrose, lactose, and gram staining kit were purchased from Sigma-Aldrich, USA. Fructooligosaccharide (FOS) was obtained from Fiatec Biosystems Sdn. Bhd, Malaysia, with a degree of polymerization between 3 and 8.

### Growth curve assay

Modified *Bifidobacterium* broth (250 µL) was dispensed in a 96-well plate in an anaerobic chamber, and the plate was then incubated and measured simultaneously inside a TECAN Spark®10 M microplate reader (TECAN, Grödig, Austria) at 37 °C for 24 h. Microbial growth was monitored by measuring the absorbance at OD 600 every 60 min intervals, with 15 s auto-shaking at 1440 rpm before each measurement. Growth Index (%) was calculated using the equation according to Bevilacqua et al. (2016), modified by Parhi et al. (2021a):

$${\text{Growth Index }}\left( \% \right) = \left\{ {\frac{{{\text{OD}}_{{{\text{MAX}}}} - {\text{OD}}_{{{\text{NC}}}} }}{{{\text{OD}}_{{{\text{PC}}}} }}} \right\} \times 100$$

where OD_MAX_ was the maximum absorbance attained, OD_NC_ was the absorbance of negative control (Modified *Bifidobacterium* broth without any sugar and FOS), and OD_PC_ was the absorbance of positive control (Modified *Bifidobacterium* broth with 4% glucose).

### Enumeration of bacteria and pH measurement

The number of viable cells in culture per mL was determined by spread plating 0.1 mL of serially diluted cultures on *Bifidobacterium* agar media and incubated at 37 °C for 48 h under anaerobic conditions. Enumeration was expressed as log CFU/mL. In addition, the pH of the samples was measured by pH-meter F-71 (LAQUA, U.S.A) after 24 h.

### Survival assay

Modified *Bifidobacterium* broth (20 mL) was dispensed in Schott bottles and inoculated at 5% w/v with *B. longum* and *B. breve*. The cultures were incubated at 37 °C under anaerobic conditions for 10 days. The cell viability (%) and pH (pH-meter F-71 (LAQUA, USA) were measured at 2-days intervals. The percentage cell viability of probiotics is calculated using the equation below (Parhi et al. 2021b):

$${\text{Cell Viability }}\left( \% \right) = {\raise0.7ex\hbox{${CFUmL_{Day - T}^{ - 1} }$} \!\mathord{\left/ {\vphantom {{CFUmL_{Day - T}^{ - 1} } {CFUmL_{Day - 0}^{ - 1} }}}\right.\kern-\nulldelimiterspace} \!\lower0.7ex\hbox{${CFUmL_{Day - 0}^{ - 1} }$}} \times 100\%$$

where *CFUmL*^−1^_*Day*−*T*_ was the viable cell count at the day of analysis and *CFUmL*^−1^_*Day*−0_ was the initial viable cell count.

### Statistical analysis

All the assays were conducted in independent triplicates. The results were statically analyzed using one-way analysis of variance (ANOVA) and Tukey’s test for post-hoc analysis. Statistical significance was determined at *p* < 0.05 using Statistical Package for the Social Sciences (SPSS) Version 23 from IBM Corporation (New York, U.S.A.).

## Results and discussion

### Growth index of *B. breve* and *B. longum* in different sugar systems with FOS supplementation

Tables 1 and 2 show the growth index (%) of *B. breve* and *B. longum* grown in modified *Bifidobacterium* medium with 2, 3, 4% glucose, fructose, sucrose, and lactose supplemented with 0.5, 1, 2, 3, 4% FOS. The growth index of *B. longum* significantly increased when the concentration of FOS, as the sole carbohydrate source, was increased from 0.5 to 4%, suggesting a dose-dependent effect of FOS on the growth (Table 1). Although a similar dose-dependent effect was observed with 0.5 and 1% FOS supplementation as a sole carbohydrate source of *B. breve*, further increase (2, 3, 4%) showed a significant decrease in growth index, which indicates partial inhibition (Table 2). The growth index of *B. longum* was 76.7% with 3% FOS while *B. breve* grew well with a growth index of 85% on 1% FOS as the sole carbohydrate source (Tables 1 and 2). The growth index > 75% stands for growth kinetics similar to that reported for the optimal conditions; growth index in the range of 25–75% underlines a partial inhibition; growth index < 25% stands for potent inhibition of the microorganism (Bevilacqua et al. 2016). These results suggest that FOS as the sole carbohydrate source supported the growth of *Bifidobacterium.* But the required concentration of FOS was different, and the growth kinetics varied according to the strain. *B. breve* ATCC 15698, 15700, and *B. longum* ATCC 15708 were reported to grow in 2% FOS as a sole carbohydrate source in de Man Rogosa Sharpe (MRS) agar (Kaplan and Hutkins 2000). *B. longum* ATCC 15707 and *B. breve* 15700 were reported to grow in 5% FOS as sole carbohydrate source (Kajiwara et al. 2002), where else *B. breve* MB 252 and *B. longum* PRO 2 were able to grow in 1% FOS as sole carbohydrate source (Rossi et al. 2005). The ability of bifidobacterial to grow on FOS might be attributed to the degree of polymerization of FOS (3 to 8), as short-chain FOS were fermented more quickly by bifidobacteria than long-chain oligosaccharides (Perrin et al. 2001; Rossi et al. 2005). Similar positive effects of short-chain FOS on the growth of bifidobacteria were reported previously. The degree of polymerization varies from 3 to 4 (Kaplan and Hutkins 2000), 3 to 8 (Perrin et al. 2001), 3–10 (Rossi et al. 2005), and 3–7 (Padalino et al. 2012). Bifidobacterial β-fructofuranosidase specifically cleaves β (2–1) bonds releasing fructose moieties in the growth medium and providing an additional carbon source for the microorganism (Ryan et al. 2005). However, the β-fructofuranosidase activity depends on the degree of polymerization and strain of bifidobacteria (Hopkins et al. 1998; Ryan et al. 2005).

**Table 1:** Growth Index (%) of *B. longum* grown in modified *Bifidobacterium* broth containing 2, 3, and 4% of glucose, fructose, sucrose, and lactose supplemented with 0.5, 1, 2, 3, and 4% of fructooligosaccharide during 24-h growth at 37 °C

		Fructooligosaccharide					
		0%	0.5%	1%	2%	3%	4%
Sugar	0%	40.06 ± 0.82^Aa^	58.16 ± 0.58^Ab^	67.45 ± 0.45^Ac^	74.86 ± 0.79^Ae^	76.72 ± 0.29^Af^	73.01 ± 0.57^Ad^
Glucose	2%	90.93 ± 0.36^Hb^	96.60 ± 0.19^Ed^	95.92 ± 0.40^Fd^	92.51 ± 0.60^BCc^	89.45 ± 0.59^Cb^	80.12 ± 1.047^Ca^
	3%	92.41 ± 0.69^HIb^	94.56 ± 0.10^Dc^	94.87 ± 0.43^Ec^	92.97 ± 0.61^BCb^	90.11 ± 0.47^Ca^	89.87 ± 0.54^Ea^
	4%	100.00 ± 0.43^Jc^	98.85 ± 0.51^FGb^	96.51 ± 0.29^FGa^	98.27 ± 0.49^Fb^	98.67 ± 0.77^Eb^	97.03 ± 0.40^Hb^
Fructose	2%	80.54 ± 0.53^Eb^	98.31 ± 0.63^Fd^	98.08 ± 0.49^GHd^	95.53 ± 0.55^DEc^	81.74 ± 0.56^Bb^	77.82 ± 1.01^Ba^
	3%	84.51 ± 0.58^Fa^	96.85 ± 0.40^Ee^	97.67 ± 0.85^Gf^	94.93 ± 0.39^Dd^	90.81 ± 0.65^BCc^	88.60 ± 0.08^Ab^
	4%	94.11 ± 0.38^Ie^	90.30 ± 0.67^Cd^	86.92 ± 0.57^Bc^	84.63 ± 0.69^Bb^	81.85 ± 0.67^Ba^	84.71 ± 0.66^Db^
Sucrose	2%	74.19 ± 0.77^ Da^	90.06 ± 0.64^Cb^	98.44 ± 1.23^He^	98.22 ± 0.25^Fe^	94.85 ± 0.61^Dd^	92.71 ± 0.51^Gc^
	3%	63.76 ± 0.76^Ca^	87.08 ± 0.52^Bb^	87.71 ± 0.66^Cb^	96.74 ± 0.56^EFe^	95.17 ± 0.28^Dd^	91.37 ± 0.33^Fc^
	4%	54.96 ± 0.50^Ba^	98.09 ± 0.91^Ed^	96.77 ± 0.35^Db^	96.01 ± 0.61^Fe^	94.56 ± 0.56^Dc^	94.99 ± 0.84^FGc^
Lactose	2%	84.12 ± 0.82^Fa^	95.93 ± 0.38^DEc^	97.61 ± 0.72^Ge^	91.86 ± 1.04^Bb^	99.60 ± 0.64^EFb^	96.77 ± 0.64^Hd^
	3%	87.09 ± 0.79^ Ga^	99.14 ± 0.62^Gf^	97.41 ± 0.42^Gd^	92.57 ± 0.11^BCc^	98.04 ± 0.99^Eef^	91.81 ± 0.36^FGb^
	4%	92.82 ± 0.77^HIa^	108.02 ± 0.74^Hc^	109.51 ± 0.75^Id^	109.87 ± 0.32^Gd^	109.13 ± 0.22^Gd^	106.72 ± 0.33^Ib^

Values are presented as means ± standard deviations (*n* = 3). ^abc^ Difference in lower case letters within a row indicates significant difference at *p* < 0.05.^ABC^ Difference in upper case letters within a column indicates significant difference at *p* < 0.05. NC: Negative control (no sugar and FOS)

**Table 2:** Growth Index (%) of *B. breve* grown in modified *Bifidobacterium* broth containing 2, 3, and 4% of glucose, fructose, sucrose, and lactose supplemented with 0.5, 1, 2, 3, and 4% of fructooligosaccharide during 24-h growth at 37 °C

		Fructooligosaccharide					
		0%	0.5%	1%	2%	3%	4%
Sugar	0%	36.33 ± 0.60^Aa^	66.61 ± 1.53^Bc^	85.02 ± 1.48^De^	76.83 ± 0.46^Dd^	65.69 ± 0.72^Ec^	55.03 ± 0.78^Db^
Glucose	2%	92.01 ± 0.41^Be^	97.20 ± 0.33^Gf^	55.48 ± 0.40^Bd^	51.54 ± 0.47^Bc^	44.07 ± 0.79^Bb^	40.39 ± 0.18^Ba^
	3%	99.21 ± 0.21^Ce^	88.60 ± 0.56^Ed^	79.21 ± 0.57^Cc^	78.80 ± 0.61^DEc^	39.04 ± 0.75^Ab^	26.74 ± 0.71^Aa^
	4%	100.00 ± 0.46^Ce^	46.33 ± 0.47^Ac^	40.62 ± 0.91^Aa^	44.57 ± 0.59^Ab^	48.94 ± 0.31^Cd^	47.38 ± 0.53^Ccd^
Fructose	2%	117.39 ± 0.77^Ge^	94.51 ± 0.36^Fd^	85.23 ± 0.81^Db^	88.20 ± 0.80^Gc^	86.58 ± 0.49^Jb^c	74.04 ± 0.83^Ha^
	3%	117.90 ± 1.22^Gf^	80.43 ± 0.10^Cd^	83.02 ± 0.79^De^	76.97 ± 0.89^DEc^	56.90 ± 0.94^Db^	48.94 ± 0.70^Ca^
	4%	94.01 ± 0.19^Bd^	85.26 ± 0.70^Dc^	82.70 ± 0.56^Dab^	81.70 ± 1.21^Fa^	81.86 ± 0.64^Iab^	83.90 ± 0.91^Jbc^
Sucrose	2%	108.83 ± 0.83^Ef^	101.92 ± 0.72^He^	89.89 ± 0.72^Ed^	77.28 ± 0.18^DEc^	71.90 ± 1.09^Fb^	62.97 ± 0.76^Fa^
	3%	104.96 ± 0.64^Df^	98.09 ± 1.82^Ge^	94.68 ± 1.06^Fd^	90.99 ± 0.52^Hc^	78.06 ± 0.34^Hb^	55.76 ± 0.78^ Da^
	4%	104.57 ± 1.73^De^	87.85 ± 0.66^Ed^	77.31 ± 1.13^Cc^	67.89 ± 1.35^Cb^	66.12 ± 0.51^Eab^	64.49 ± 0.27^FGa^
Lactose	2%	101.20 ± 0.30^Cde^	103.23 ± 0.79^He^	100.89 ± 0.84^Gd^	97.63 ± 1.38^Jc^	74.63 ± 0.72^ Gb^	66.50 ± 0.31^ Ga^
	3%	113.54 ± 0.41^Fe^	106.35 ± 0.85^Id^	105.19 ± 0.94^Hd^	95.09 ± 0.49^Ic^	84.59 ± 0.30^Jb^	79.67 ± 0.94^Ia^
	4%	110.23 ± 0.88^Ee^	117.53 ± 0.42^Jf^	105.09 ± 0.49^Hd^	79.33 ± 0.74^EFc^	76.89 ± 0.61^Hb^	58.05 ± 0.97^Ea^

Values are presented as means ± standard deviations (*n* = 3). ^abc^ Difference in lower case letters within a row indicates significant difference at *p* < 0.05.^ABC^ Difference in upper case letters within a column indicates significant difference at *p* < 0.05. NC: Negative control (no sugar and FOS)

The percentage increase was determined by comparing the growth index of the FOS supplemented sugar system with the growth index of the non-supplemented respective sugar system. The highest percentage of increase in growth index, 78.5% of *B. longum,* was observed with 4% sucrose supplemented with 0.5% FOS (Table 1). The supplementation of 0.5–4% FOS resulted in the percentage increase in growth index of 21.5–78.3% (Table 1) of *B. longum* along with a significant increase in cell density to approximately 9.36–10.47 log CFU/mL (Table 3) in the presence of 2, 3, 4% sucrose, suggesting a growth-promoting effect of FOS (Table 1). *B. longum* also showed a significant increase in cell density to approximately 9.36–10.47 log CFU/mL (Table 3) correlating with a significant decrease in pH (4.98–6.00) in media containing both sucrose and FOS compared to non-supplemented media (Table S1, supplementary data). Yeo and Liong (2010) reported positive effects of FOS supplementation on the growth of *B. longum* FTDC 8643 in soymilk. Sucrose is the predominant sugar in soymilk (Turroni et al. 2011). The FOS supplementation significantly improved the growth index of *B. longum* in the presence of sucrose (Table 1) because *B. longum* comprises multiple sugar transport systems and an additional pathway known as a fructose-6-phosphate shunt or ‘bifid’ shunt (De Vries and Stouthamer 1967), and the ability to produce carbohydrate modifying enzymes. According to Kullin et al. (2006), the *cscA* (β-fructofuranosidase) and *scrP* (sucrose phosphorylase) gene clusters of *B. longum* NCIMB 702259 function mainly in the metabolism of intracellular sucrose generated from the breakdown of more complex carbohydrates. The *scrP* gene was up-regulated in the presence of sucrose relative to glucose (Kullin et al. 2006). Henceforth, the growth-promoting effect of FOS in the presence of sucrose may be due to *B. longum* utilizing sucrose and FOS simultaneously.

**Table 3:** Viable count (log CFU/mL) of *B. longum* grown in modified *Bifidobacterium* broth containing 2, 3, and 4% of glucose, fructose, sucrose, and lactose supplemented with 0.5, 1, 2, 3 and 4% of fructooligosaccharide during 24-h growth at 37 °C

		Fructooligosaccharide					
		0%	0.5%	1%	2%	3%	4%
Sugar	0%	6.03 ± 0.51^Aa^	7.97 ± 0.01^Ab^	8.20 ± 0.01^Ab^	8.21 ± 0.01^Ab^	8.28 ± 0.00^Bb^	8.21 ± 0.01^Bb^
Glucose	2%	8.20 ± 0.03^Ca^	9.19 ± 0.04^BCb^	10.13 ± 0.04^Dc^	10.32 ± 0.02^Ed^	9.15 ± 0.02^Db^	8.20 ± 0.05^Ba^
	3%	8.24 ± 0.04^Ca^	9.27 ± 0.02^CDc^	9.09 ± 0.04^Bb^	9.28 ± 0.03^Cc^	9.16 ± 0.01^Dbc^	8.22 ± 0.03^Ba^
	4%	8.06 ± 0.08^Ca^	10.31 ± 0.04^Gd^	10.12 ± 0.03^Dc^	9.27 ± 0.08^Cb^	8.20 ± 0.06^Ba^	8.18 ± 0.01^Ba^
Fructose	2%	8.15 ± 0.03^Ca^	9.09 ± 0.11^Bb^	10.12 ± 0.08^Dc^	10.18 ± 0.03^Dc^	8.01 ± 0.04^Aa^	8.00 ± 0.02^Aa^
	3%	8.13 ± 0.04^Ca^	9.12 ± 0.02^Bc^	9.04 ± 0.08^Bbc^	9.15 ± 0.06^Bc^	8.95 ± 0.06^Cbc^	8.90 ± 0.08^Cb^
	4%	8.14 ± 0.02^Cb^	10.05 ± 0.01^Fe^	9.02 ± 0.05^Bc^	8.30 ± 0.01^Ac^	7.97 ± 0.03^Aa^	8.00 ± 0.02^Aa^
Sucrose	2%	7.05 ± 0.02^Ba^	9.34 ± 0.03^DEc^	10.47 ± 0.02^Ee^	10.33 ± 0.00^Ed^	9.34 ± 0.02^Ec^	8.30 ± 0.03^Bb^
	3%	7.00 ± 0.02^Ba^	9.47 ± 0.03^Ec^	9.36 ± 0.01^Cb^	10.33 ± 0.02^Ed^	10.43 ± 0.01^Ge^	9.41 ± 0.01^Dbc^
	4%	7.62 ± 0.03^Ba^	10.36 ± 0.04^Gcd^	10.39 ± 0.01^Ed^	10.36 ± 0.01^Ecd^	10.26 ± 0.01^Fb^	10.30 ± 0.02^Ebc^
Lactose	2%	8.31 ± 0.03^Ca^	9.33 ± 0.02^DEb^	10.35 ± 0.00^Ec^	10.37 ± 0.02^Ec^	9.33 ± 0.03^Eb^	8.29 ± 0.02^Ba^
	3%	8.28 ± 0.03^Ca^	9.31 ± 0.01^Db^	9.33 ± 0.02^Cb^	10.35 ± 0.01^Ec^	10.31 ± 0.01^Fc^	9.27 ± 0.03^Db^
	4%	8.34 ± 0.04^Ca^	10.36 ± 0.01^Gcd^	10.38 ± 0.01^Ed^	10.36 ± 0.01^Ec^	10.32 ± 0.02^Fb^	10.46 ± 0.00^Ee^

Values are presented as means ± standard deviations (*n* = 3). ^abc^ Difference in lower case letters within a row indicates significant difference at *p* < 0.05.^ABC^ Difference in upper case letters within a column indicates significant difference at *p* < 0.05. NC: Negative control (no sugar and FOS)

In the lactose system, *B. longum* showed the highest percentage of increase in growth index of 18.4% in 4% lactose with 2% FOS supplementation (Table 1). A significant increase in the cell density to approximately 8.29–10.46 log CFU/mL (Table 3) was observed in a medium containing lactose with FOS supplementation compared to the non-supplemented lactose system. *B. breve* showed the significant highest increase of 6.6% in the growth index in 4% lactose supplemented with 0.5% FOS, suggesting a growth-promoting effect at a low concentration of FOS in the lactose system (Table 2). Similarly, the supplementation of FOS was the most effective in enhancing the growth rate of both *B. bifidum* Bf-1 and Bf-6 in skim milk (Shin et al. 2000) and induced a higher growth rate in *B.* *adolescentis* and *B.* *catenulatum* in milk (Padalino et al. 2012). FOS supplementation also showed the most increase in the growth-promoting activity for *B. breve* 3022 and *B. longum* 3128 in skim milk (Choi and Shin 2006). Lactose is the predominant sugar in milk-based products. Most of the predominant bifidobacterial species in infants' intestines, *B. longum*, *B. breve*, and *B. bifidum*, produce galacto-*N*-biose/lacto-*N*-biose I phosphorylase responsible for lacto-*N*-biose degradation (Turroni et al. 2011; Xiao et al. 2010). In addition, Parche et al (2006) reported that putative glucose transporter gene *glcP* in *B. longum* NCC2705 is repressed by lactose when grown in a medium containing both lactose and glucose. Therefore, *B. longum* and *B. breve* might be using both lactose and FOS simultaneously, resulting in a positive effect of FOS in the lactose system.

In glucose and fructose systems, 1.7–6.2% and 4.8–22.1% increase in growth index was observed in 2 and 3% glucose and fructose with 0.5–2% and 0.5–4% FOS supplementation, respectively, suggesting a positive effect of FOS supplementation on the growth of *B. longum* in lower concentrations of glucose and fructose (Table 1). Interestingly, *B. breve* showed a 5.6% increase in growth index in the presence of 2% glucose supplemented with 0.5% FOS (Table 2). At the same time, other combinations showed partial inhibition of *B. longum* and *B. breve* (Tables 1 and 2). For example, Parche et al. (2007) reported the sugar transport system of *B. longum* NCC 2705, which included ABC, PEP–PTS, major intrinsic protein family (MIP), Major facilitator superfamily (MFS), and glycoside-pentoside-hexuronide cation symporter family (GPH). These multiple sugar transport systems in *B. longum* give it the ability to uptake a wide range of carbohydrates, including polymers such as FOS, which can be the reason behind the positive effect of FOS supplementation on the growth index of *B. longum* in all four sugars (Table 1). However, the operon in *B. breve* UCC2003, which can break down FOS, was activated when grown in sucrose but repressed when grown in glucose, fructose, or combinations of glucose-sucrose, fructose-sucrose (Ryan et al. 2005). This might be occurring here, resulting in significantly lower growth index values (*p* < 0.05) of *B. breve* grown in several media containing glucose, fructose, and lactose with FOS supplementation as compared to the positive control of glucose without supplementation (Table 2).

*B. longum* comprises multiple sugar transport systems (De Vries and Stouthamer 1967) and can produce carbohydrate modifying enzymes. According to Pokusaeva et al (2011), the *Bifidobacterium* genome reflects the metabolic adaptation to a complex carbohydrate-rich gastrointestinal tract environment as it encodes a large number of predicted carbohydrate-modifying enzymes. Therefore, *B. longum* can utilize different carbohydrates resulting in a positive effect on the growth index with FOS supplementation. Similarly, a positive effect on the log CFU/mL of *B. longum* was observed, resulting in a 2–3 log increase in log CFU/mL in the sucrose system and 1–2 log increase in log CFU/mL in glucose, fructose, lactose systems with FOS supplementation compared to respective sugar systems without FOS supplementation (Table 3). Nevertheless, *B. breve* showed no log CFU/mL increase with FOS supplementation in sugar systems (Table 4). The sharp decrease in pH in media might explain the difference in log CFU/mL of *B. breve* except for the lactose system (Table S2, supplementary data). This is most likely due to the growth index being calculated as the total sum of all the OD taken every hour, while log CFU/mL was calculated by taking CFU initially and at the end of 24 h. However, *B. breve* and *B. longum* entered the initial death phase due to depletion of carbon source, media acidification, and accumulation of organic acids, resulting in a decrease in CFU at the end. Nevertheless, microbial growth and survival during the production and storage of fermented foods are subjected to several abiotic stresses such as acidification of media, nutritional availability, and accumulation of byproducts and dead cells in ferment media strongly depend on the cells to adapt.

**Table 4:** Viable count (log CFU/mL) of *B. breve* grown in modified *Bifidobacterium* broth containing 2, 3, and 4% of glucose, fructose, sucrose, and lactose supplemented with 0.5, 1, 2, 3 and 4% of fructooligosaccharide during 24-h growth at 37 °C

		Fructooligosaccharide					
		0%	0.5%	1%	2%	3%	4%
Sugar	0%	6.19 ± 0.00^Aa^	7.21 ± 0.03^Ab^	7.46 ± 0.45^Ab^	7.08 ± 0.01^Ab^	7.07 ± 0.00^Ab^	7.11 ± 0.12^Ab^
Glucose	2%	8.12 ± 0.04^BCa^	8.13 ± 0.04^Ba^	8.32 ± 0.00^BCb^	8.29 ± 0.02^EFb^	8.32 ± 0.00^CDb^	8.29 ± 0.01^Cb^
	3%	8.20 ± 0.02^BCa^	8.29 ± 0.05^BCab^	8.29 ± 0.00^BCbc^	8.27 ± 0.01^EFbc^	8.32 ± 0.01^CDc^	8.29 ± 0.01^Cbc^
	4%	8.44 ± 0.00^Dd^	8.32 ± 0.01^BCc^	8.27 ± 0.01^BC^b	8.30 ± 0.00^EFbc^	8.16 ± 0.02^Ca^	8.28 ± 0.00^Bb^
Fructose	2%	7.99 ± 0.01^Bab^	7.97 ± 0.04^Ba^	8.05 ± 0.02B^Cabc^	8.01 ± 0.07^Babc^	8.08 ± 0.01^Bbc^	8.11 ± 0.01^Bc^
	3%	8.00 ± 0.04^Ba^	8.00 ± 0.08^Ba^	7.96 ± 0.02^Ba^	8.07 ± 0.01^BCa^	8.08 ± 0.02^Ba^	8.09 ± 0.01^Ba^
	4%	8.15 ± 0.03^BCc^	8.07 ± 0.02^Bab^	8.02 ± 0.02^BCa^	8.12 ± 0.02^BCDbc^	8.06 ± 0.01^Bab^	8.09 ± 0.03^Babc^
Sucrose	2%	8.03 ± 0.04^Bab^	8.16 ± 0.03^BCbc^	8.17 ± 0.02^BCc^	8.21 ± 0.07^DEc^	8.21 ± 0.02^Cc^	8.02 ± 0.03^Ba^
	3%	8.17 ± 0.21^BCa^	8.61 ± 0.47^Ca^	8.10 ± 0.02^BCa^	8.18 ± 0.08^CDEa^	8.28 ± 0.03^ Da^	8.14 ± 0.11^Ca^
	4%	8.19 ± 0.01^BCb^	8.30 ± 0.00^BCc^	8.10 ± 0.03^BCa^	8.32 ± 0.01^EFc^	8.30 ± 0.01^CDc^	8.34 ± 0.01^Cc^
Lactose	2%	8.30 ± 0.00^CDa^	8.36 ± 0.01^BCb^	8.35 ± 0.03^BCb^	8.35 ± 0.01^Fb^	8.30 ± 0.00^CDa^	8.35 ± 0.02^Cb^
	3%	8.28 ± 0.02^CDa^	8.33 ± 0.01^BCbc^	8.34 ± 0.00^Cc^	8.30 ± 0.00^EFab^	8.32 ± 0.02^CDabc^	8.34 ± 0.01^Cbc^
	4%	8.34 ± 0.01^CDb^	8.38 ± 0.01^BCc^	8.36 ± 0.00^Cbc^	8.30 ± 0.00^EFa^	8.34 ± 0.01^Eb^	8.36 ± 0.02^Cbc^

Values are presented as means ± standard deviations (*n* = 3). ^abc^ Difference in lower case letters within a row indicates significant difference at *p* < 0.05.^ABC^ Difference in upper case letters within column indicates a significant difference at *p* < 0.05. NC: Negative control (no sugar and FOS)

### Survival of *B. longum* and *B. breve* in different sugar systems with FOS supplementation

**Fig. 1:**
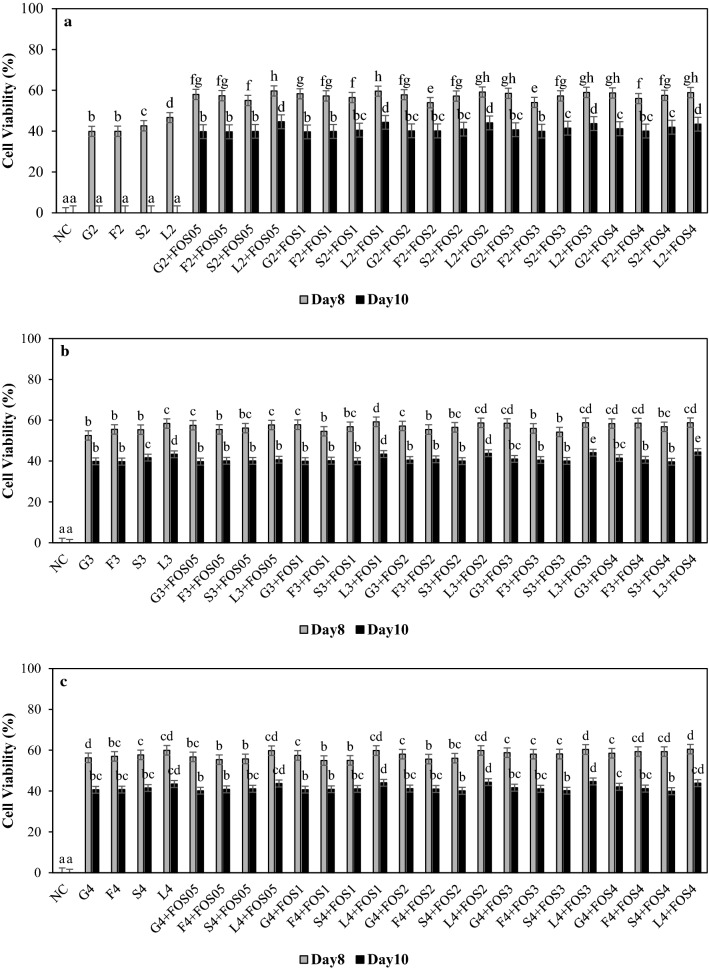
Cell viability (%) of *B. longum* grown in modified *Bifidobacterium* broth containing **a** 2% glucose (G2), fructose (F2), sucrose (S2), lactose (L2), **b** 3% glucose (G3), fructose (F3), sucrose (S3), lactose (L3), and (**c**) 4% glucose (G4), fructose (F4), sucrose (S4), lactose (L4) with 0.5% (FOS05), 1% (FOS1), 2% (FOS2), 3% (FOS3) and 4% (FOS4) fructooligosaccharide on 8th and 10th day. ^abc^ Difference in lower case letters indicates significant differences between different treatments within a same day at *p* < 0.05. NC = negative control (no sugar and FOS)

**Fig. 2:**
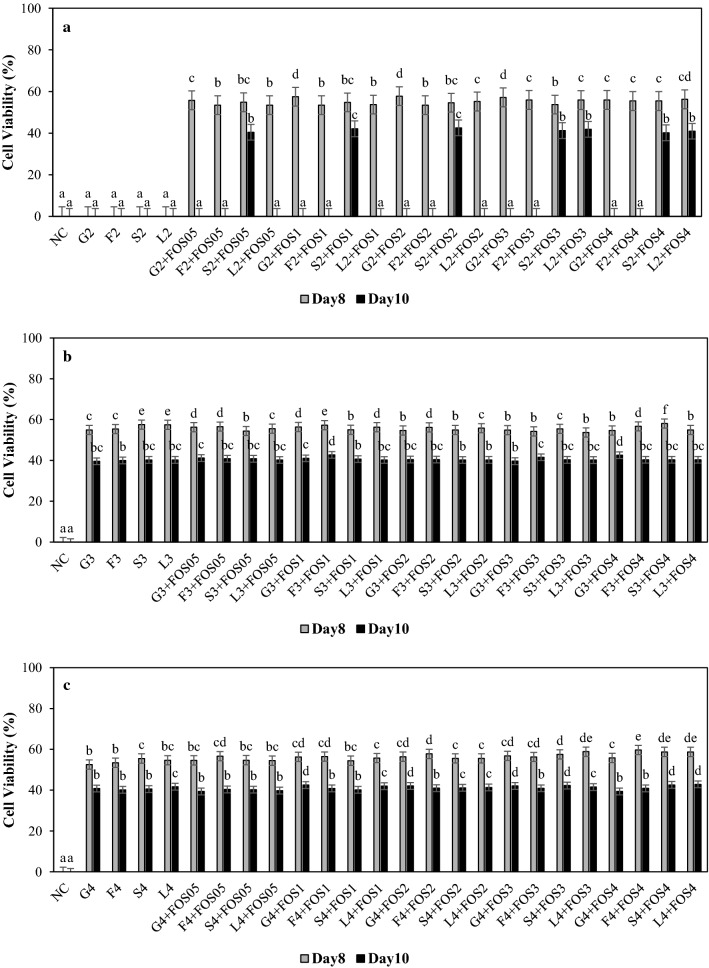
Cell viability (%) of *B. breve* grown in modified *Bifidobacterium* broth containing **a**)2% glucose (G2), fructose (F2), sucrose (S2), lactose (L2), **b** 3% glucose (G3), fructose (F3), sucrose (S3), lactose (L3), and **c** 4% glucose (G4), fructose (F4), sucrose (S4), lactose (L4) with 0.5% (FOS05), 1% (FOS1), 2% (FOS2), 3% (FOS3) and 4% (FOS4) fructooligosaccharide on 8th and 10th day. ^abc^ Difference in lower case letters indicates significant differences between different treatments within a same day at *p* < 0.05. NC = negative control (no sugar and FOS)

The survival assay focused on the effects of FOS on the death kinetics of *B. breve* and *B. longum* at 37˚C. As expected, the cell viability of *B. longum* and *B. breve* increased until day-2 of the assay. However, a decrease in cell viability of *B. longum* and *B. breve* was observed with cell viability of 68.6–73.4% and 64.7–72.8% on day-6, suggesting both the microorganism showed prolonged viability in sugar systems with and without FOS supplementation, respectively (data not shown). The changes in survival and pH of *B. longum* and *B. breve* in various sugar systems with FOS supplementation on day-8 and day-10 are presented in Figs. 1, 2, and Figs. S1, S2 (supplementary data). These 2 days were selected to evaluate the difference between the survival of *B. breve* and *B. longum* in sugar systems with and without FOS supplementation at the death phase. The decline in cell viability after day-2 and no change in pH after day-6 suggested media saturation and acidification (data not shown). In the presence of 2% sugar systems without FOS supplementation, the cell viability of *B. longum* and *B. breve* was between 39–46% and 0% on day-8, respectively. However, 0% cell viability was observed for both *B. longum* and *B. breve* in 2% sugar systems without FOS supplementation (Figs. 1a and 2a). Similarly, Akalin et al. (2004) reported a decrease in the cell viability and pH of *B. longum* after seven days of refrigerated storage, concluding that the low pH was the critical factor in the viability of bifidobacterial cells. Acetic acid and lactic acid, byproducts of bifidobacteria, are known as environmental stress that may inhibit the growth of microorganisms by entering the cell in its non-dissociated form and then dissociating within the cell, which causes acidification of the cytoplasm, collapsing of the proton motive force, and inhibition of the enzyme reactions, resulting in the inhibition of nutrient transport (Guan and Liu 2020). Although pH was below 4.0 (Figs. S1 and S2, supplementary data), both *B. longum* and *B. breve* showed 39–44% cell viability in 3 and 4% sugar systems without FOS supplementation. This could be due to the ability of bifidobacteria to produce exopolysaccharides (EPS) under stressful conditions, thereby providing tolerance against acidic pH, contributing to cell protection and survival (Alp and Aslim 2010; Fanning et al. 2012). Audy et al (2010) suggested that EPS production by *B. longum* BB79 and *B. longum* CRC002 were induced by lactose, fructose, and glucose. *B. breve* UCC2003 and *B. breve* DSM20213 were reported to produce exopolysaccharides, thus increasing stress tolerance against low pH (Alp and Aslim 2010; Fanning et al. 2012).

The 50% cell viability of *B. longum* and *B. breve* in 2% sugar systems with FOS supplementation on day-8 suggests FOS supplementation resulted in slower death kinetic (Fig. 1a and 2a). *B. longum* showed the highest cell viability of 43–44% in 2% lactose with FOS supplementation on day-10. However, *B. breve* showed the highest cell viability of 42% in the 2% sucrose system with 1 and 2% FOS supplementation followed by 2% sucrose system with 0.5, 3, 4% FOS supplementation, and 2% lactose with 3 and 4% FOS supplementation on day-10 (Fig. 2a). These results suggest the positive effect of FOS supplementation on the cell viability of *B. longum* in all sugar systems till day-10. However, the cell viability of *B. breve* was only observed in 2% sucrose with 0.5-4% FOS supplementation, and 2% lactose with 3 and 4% FOS supplementation on day-10 (Fig. 2a). Nevertheless, the difference in survival of *B. longum* and *B. breve* suggests the diverse nature of *Bifidobacterium* to utilize simple and complex carbohydrates, which also can vary between strains and species (Parche et al. 2007).

In higher concentrations, 3 and 4% sugar systems, the FOS supplementation positively influenced cell viability of *B. longum,* resulting in 39–44% cell viability on day-10 (Fig. 1b and c). Similarly, *B. breve* showed 39–42% cell viability in 3 and 4% sugar systems with FOS supplementation on day-10 (Fig. 2b and 2c). These results are consistent with previous reports on the ability of FOS to stimulate the viability of *Bifidobacterium* in different mediums. For example, FOS supplementation in skim milk was effective in increasing the viability of *B. bifidum* Bf-1 and Bf-6 after four weeks at 4 °C (Shin et al. 2000), and viability of *B. longum* was significantly higher with FOS supplementation in skim milk at 4 °C for four weeks storage (Choi and Shin 2006). In addition, Akalin et al. (2004) reported that *B. longum* maintained viability above 10^6^ CFU/g in yoghurt containing FOS for up to 21 days at 4 °C.

## Conclusion

The present study shows that FOS supplementation significantly increased the growth index of *B. longum* in most sugar systems, with few exceptions. However, only 2% glucose and 4% lactose supplemented with 0.5% FOS supplementation showed a significant increase in the growth index of *B. breve*. Based on the highest percentage increase in growth index, sucrose was the most suitable sugar for FOS supplementation for *B. longum,* whereas lactose was the most suitable sugar for FOS supplementation for *B. breve*. Furthermore, in the survival assay, FOS supplementation in 2% sugar systems effectively influenced the cell viability and slowed the death phase of *B. longum* and *B. breve*. The highest cell viability of *B. longum* was observed in 2% lactose with FOS supplementation. On the other hand, the highest cell viability of *B. breve* was observed in 2% sucrose with FOS supplementation. Thus, the growth and survival of *B. longum* and *B. breve* were improved and prolonged with FOS supplementation, but the effect was depended on the concentration and type of sugar system and FOS concentration. This study demonstrated that the use of sugar systems without the complexity of a food matrix provided valuable information to understand the effect of FOS supplementation on the growth and survival of *B. longum* and *B. breve.* However, as there are other components in a food matrix that may influence the growth and survival of *B. longum* and *B. breve,* the results in this study cannot be directly applied to a food matrix. Nevertheless, these results provide insights into developing efficient and improved synbiotic products containing bifidobacteria with FOS supplementation.

## Supplementary Information

Below is the link to the electronic supplementary material.

Supplementary file1 (DOCX 71 kb)

## Data Availability

Not applicable.
