# Supplementary material for: Growth and survival of Bifidobacterium breve and Bifidobacterium longum in various sugar systems with fructooligosaccharide supplementation
Source: J Food Sci Technol. 2022 Jan 20;59(10):3775–86. doi: 10.1007/s13197-022-05361-z (PMC9525548; doi:10.1007/s13197-022-05361-z)
Supplement: Supplementary file 1 — Supplementary file1 (DOCX 71 kb) [file 13197_2022_5361_MOESM1_ESM.docx]

**Supplementary Material**

**Table S1** pH of *B. longum* grown in modified *Bifidobacterium* broth containing 2, 3, and 4% of glucose, fructose, sucrose, and lactose supplemented with 0.5, 1, 2, 3 and 4% of fructooligosaccharide during 24-h growth at 37⁰C

|  |  | **Fructooligosaccharide** | | | | | |
| --- | --- | --- | --- | --- | --- | --- | --- |
|  |  | **0%** | **0.5%** | **1%** | **2%** | **3%** | **4%** |
| **Sugar** | **0%** | 6.80 ± 0.20^Ec^ | 5.64 ± 0.12^Ea^ | 5.73 ± 0.12^CDab^ | 6.37 ± 0.37^Dbc^ | 6.76 ± 0.19^Dc^ | 6.96 ± 0.10^Dc^ |
| **Glucose** | **2%** | 4.86 ± 0.13^BCa^ | 5.11 ± 0.15^BCDab^ | 5.23 ± 0.24^ABCabc^ | 5.56 ± 0.16^ABCDbc^ | 5.67 ± 0.11^Cc^ | 5.74 ± 0.13^ABc^ |
|  | **3%** | 4.54 ± 0.12^Ba^ | 4.72 ± 0.02^ABa^ | 4.78 ± 0.00^ABa^ | 5.14 ± 0.13^ABb^ | 5.29 ± 0.13^ABCbc^ | 5.52 ± 0.04^ABc^ |
|  | **4%** | 4.03 ± 0.01^Aa^ | 4.40 ± 0.04^Aab^ | 4.66 ± 0.15^Abc^ | 4.74 ± 0.14^Abcd^ | 4.90 ± 0.10^Acd^ | 5.11 ± 0.15^Ad^ |
| **Fructose** | **2%** | 5.06 ± 0.02^CDa^ | 5.12 ± 0.02^BCDab^ | 5.23 ± 0.08^ABCbc^ | 5.38 ± 0.04^ABCDcd^ | 5.44 ± 0.01^BCef^ | 5.54 ± 0.06^ABf^ |
|  | **3%** | 5.03 ± 0.01^CDa^ | 5.06 ± 0.02^BCDa^ | 5.15 ± 0.12^ABCab^ | 5.26 ± 0.14^ABCabc^ | 5.42 ± 0.09^ABCbc^ | 5.48 ± 0.10^ABc^ |
|  | **4%** | 5.20 ± 0.10^CDa^ | 5.30 ± 0.10^CDEab^ | 5.55 ± 0.00^BCDab^ | 5.83 ± 0.22^BCDb^ | 6.60 ± 0.35^Dc^ | 6.86 ± 0.14^Dc^ |
| **Sucrose** | **2%** | 4.87 ± 0.10^BCa^ | 4.98 ± 0.07^BCab^ | 5.04 ± 0.01^ABCab^ | 5.12 ± 0.00^ABbc^ | 5.13 ± 0.01^ABbc^ | 5.25 ± 0.07^ABc^ |
|  | **3%** | 4.89 ± 0.10^BCa^ | 4.99 ± 0.08^BCab^ | 5.06 ± 0.01^ABCab^ | 5.15 ± 0.11^ABabc^ | 5.26 ± 0.10^ABCbc^ | 5.42 ± 0.09^ABc^ |
|  | **4%** | 5.11 ± 0.09^CDa^ | 5.32 ± 0.00^CDEab^ | 5.47 ± 0.09^BCDab^ | 5.50 ± 0.09^ABCDab^ | 5.60 ± 0.06^BCab^ | 6.00 ± 0.61^BCb^ |
| **Lactose** | **2%** | 5.37 ± 0.25^Da^ | 5.50 ± 0.29^DEa^ | 6.07 ± 0.67^Da^ | 6.29 ± 0.67^CDa^ | 6.73 ± 0.16^Da^ | 6.81 ± 0.20^Da^ |
|  | **3%** | 5.05 ± 0.01^CDa^ | 5.18 ± 0.09^CDa^ | 5.20 ± 0.09^ABCa^ | 5.32 ± 0.16^ABCa^ | 6.53 ± 0.01^Db^ | 6.72 ± 0.14^CDb^ |
|  | **4%** | 5.21 ± 0.22^CDa^ | 5.24 ± 0.23^CDEa^ | 5.37 ± 0.23^ABCDa^ | 5.74 ± 0.55^ABCDa^ | 6.75 ± 0.14^Db^ | 6.78 ± 0.14^Db^ |

Values are presented as means ± standard deviations (n=3). ^abc^ Difference in lower case letters within a row indicates significant difference at *p* < 0.05.^ABC^ Difference in upper case letters within a column indicates significant difference at *p* < 0.05. NC: Negative control (no sugar and FOS).

**Table S2** pH of *B. breve* grown in modified *Bifidobacterium* broth containing 2, 3 and 4% of glucose, fructose, sucrose, and lactose supplemented with 0.5, 1, 2, 3 and 4% of fructooligosaccharide during 24-h growth at 37⁰C

|  |  | **Fructooligosaccharide** | | | | | |
| --- | --- | --- | --- | --- | --- | --- | --- |
|  |  | **0%** | **0.5%** | **1%** | **2%** | **3%** | **4%** |
| **Sugar** | **0%** | 6.86 ± 0.11^Eb^ | 6.42 ± 0.43^Db^ | 5.31 ± 0.20^Ea^ | 4.94 ± 0.15^Cba^ | 5.03 ± 0.02^Da^ | 5.06 ± 0.04^CDa^ |
| **Glucose** | **2%** | 4.16 ± 0.20^ABa^ | 4.27 ± 0.19^ABa^ | 4.43 ± 0.05^Ba^ | 4.48 ± 0.04^ABa^ | 4.55 ± 0.13^Ba^ | 4.56 ± 0.12^ABa^ |
|  | **3%** | 4.40 ± 0.05^ABCa^ | 4.45 ± 0.01^ABCa^ | 4.49 ± 0.05^BCDa^ | 4.64 ± 0.12^BCab^ | 4.67 ± 0.14^BCab^ | 4.87 ± 0.19^ABCb^ |
|  | **4%** | 4.55 ± 0.11^BCa^ | 4.71 ± 0.03^BCab^ | 4.78 ± 0.07^Dab^ | 4.84 ± 0.12^Cab^ | 4.94 ± 0.12^CDb^ | 5.01 ± 0.16^BCb^ |
| **Fructose** | **2%** | 4.44 ± 0.01^ABCa^ | 4.45 ± 0.01^ABCa^ | 4.45 ± 0.01^BCa^ | 4.72 ± 0.00^BCb^ | 4.76 ± 0.01^BCDb^ | 5.00 ± 0.17^BCc^ |
|  | **3%** | 4.45 ± 0.02^ABCa^ | 4.56 ± 0.09^ABCab^ | 4.64 ± 0.12^BCDb^ | 4.75 ± 0.02^BCbc^ | 4.79 ± 0.00^BCDcd^ | 4.85 ± 0.05^ABCd^ |
|  | **4%** | 4.17 ± 0.18^ABa^ | 4.41 ± 0.01^ABCab^ | 4.44 ± 0.01^BCab^ | 4.48 ± 0.05^ABab^ | 4.71 ± 0.03^BCb^ | 4.85 ± 0.05^ABC^ |
| **Sucrose** | **2%** | 4.72 ± 0.01^Ca^ | 4.98 ± 0.14^Cab^ | 5.17 ± 0.15^Ebc^ | 5.27 ± 0.15^Dbc^ | 5.42 ± 0.09^Ec^ | 5.49 ± 0.09^Dc^ |
|  | **3%** | 4.06 ± 0.01^Aa^ | 4.52 ± 0.30^ABCab^ | 4.76 ± 0.03^CDb^ | 4.80 ± 0.04^BCb^ | 4.94 ± 0.13^CDb^ | 4.99 ± 0.14^BCb^ |
|  | **4%** | 4.03 ± 0.00^Aa^ | 4.04 ± 0.01^Aa^ | 4.08 ± 0.03^Aa^ | 4.17 ± 0.11^Aa^ | 4.22 ± 0.16^Aa^ | 4.43 ± 0.25^Aa^ |
| **Lactose** | **2%** | 5.26 ± 0.15^Da^ | 6.54 ± 0.02^Db^ | 6.64 ± 0.10^Gbc^ | 6.72 ± 0.05^Fbc^ | 6.82 ± 0.01^Gc^ | 6.86 ± 0.03^Dc^ |
|  | **3%** | 5.42 ± 0.26^Da^ | 6.34 ± 0.14^Db^ | 6.49 ± 0.02^Gb^ | 6.53 ± 0.03^EFb^ | 6.58 ± 0.05^FGb^ | 6.64 ± 0.12^Db^ |
|  | **4%** | 5.54 ± 0.06^Da^ | 6.13 ± 0.01^Db^ | 6.15 ± 0.01^Fb^ | 6.26 ± 0.12^Ebc^ | 6.43 ± 0.01^Fcd^ | 6.47 ± 0.04^Dd^ |

Values are presented as means ± standard deviations (n=3).^abc^ Difference in lower case letters within a row indicates significant difference at *p* < 0.05.^ABC^ Difference in upper case letters within a column indicates significant difference at *p* < 0.05. NC: Negative control (no sugar and FOS).

|  |
| --- |
|  |
|  |

**Figure S1.** pH of *B. longum* grown in modified *Bifidobacterium* broth media containing (**a**) 2% glucose (G2), fructose (F2), sucrose (S2), lactose (L2), (**b**) 3% glucose (G3), fructose (F3), sucrose (S3), lactose (L3), and (**c**) 4% glucose (G4), fructose (F4), sucrose (S4), lactose (L4) with 0.5% (FOS05), 1% (FOS1), 2% (FOS2), 3% (FOS3) and 4% (FOS4) fructooligosaccharide on 8^th^ and 10^th^ day. ^abc^Difference in lower case letters indicates significant differences between different treatments within a same day at p < 0.05. NC = negative control (no sugar and FOS).

|  |
| --- |
|  |
|  |

**Figure S2.** pH of *B. breve* grown in modified *Bifidobacterium* broth media containing (**a**) 2% glucose (G2), fructose (F2), sucrose (S2), lactose (L2), (**b**) 3% glucose (G3), fructose (F3), sucrose (S3), lactose (L3), and (**c**) 4% glucose (G4), fructose (F4), sucrose (S4), lactose (L4) with 0.5% (FOS05), 1% (FOS1), 2% (FOS2), 3% (FOS3) and 4% (FOS4) fructooligosaccharide on 8^th^ and 10^th^ day. ^abc^Difference in lower case letters indicates significant differences between different treatments within a same day at p < 0.05. NC = negative control (no sugar and FOS).
